# EDPNet (Efficient DB and PARSeq Network): A Robust Framework for Online Digital Meter Detection and Recognition Under Challenging Scenarios

**DOI:** 10.3390/s25082603

**Published:** 2025-04-20

**Authors:** Songwen Guan, Zhitian Niu, Ming Kong, Shiling Wang, Hangbo Hua

**Affiliations:** College of Metrology Measurement and Instrument, China Jiliang University, Hangzhou 310048, China; guansongwen@cjlu.edu.cn (S.G.); slwang@cjlu.edu.cn (S.W.); hangbo.h@cjlu.edu.cn (H.H.)

**Keywords:** deep learning, digital meter reading, EDPNet, sensor data processing, smart sensor systems

## Abstract

Challenges such as perspective distortion, irregular reading regions, and complex backgrounds in natural scenes hinder the accuracy and efficiency of automatic meter reading systems. Current mainstream approaches predominantly utilize object-detection-based methods without optimizing for text characteristics, while enhancements in detection robustness under complex backgrounds typically focus on data preprocessing rather than model architecture. To address these limitations, a novel end-to-end framework, i.e., EDPNet (Efficient DB and PARSeq Network), is proposed to integrate efficient boundary detection and text recognition. EDPNet comprises two key components, EDNet for detection and EPNet for recognition, where EDNet employs EfficientNetV2-s as its backbone with the Multi-Scale KeyDrop Attention (MSKA) and Efficient Multi-scale Attention (EMA) mechanisms to address perspective distortion and complex background challenges, respectively. During the recognition stage, EPNet integrates a DropKey Attention module into the PARSeq encoder, enhancing the recognition of irregular readings while effectively mitigating overfitting. Experimental evaluations show that EDNet achieves an F1-score of 0.997988, outperforming DBNet++ (ResNet50) by 0.61%. In challenging scenarios, EDPNet surpasses state-of-the-art methods by 0.7~1.9% while reducing parameters by 20.03%. EPNet achieves 90.0% recognition accuracy, exceeding the current best performance by 0.2%. The proposed framework delivers superior accuracy and robustness in challenging conditions while remaining lightweight.

## 1. Introduction

Accurate and efficient digital meter reading is fundamental to the metering and management of utilities such as water, electricity, and gas [1]. Traditional manual meter reading methods are labor-intensive and time-consuming, requiring significant on-site effort, which makes them highly inefficient and unsuitable for real-time monitoring. Furthermore, these methods are prone to inaccuracies caused by human error or subjective judgment, leading to potential billing disputes. Although smart meters capable of remote automatic reading and data transmission have been widely adopted in developed regions [2], improving both efficiency and accuracy, their large-scale deployment remains impractical in many underdeveloped areas due to cost constraints [3]. As an alternative, automatic meter reading (AMR) technologies that take advantage of mobile devices or fixed cameras have recently gained traction for their cost-effectiveness and efficiency. However, these systems face several challenges in real-world applications, including pixelation, foggy blur, and extreme illumination variations of images. Limitations in shooting angles and distances often result in perspective distortions and deformations in the captured meter region as well, further complicating the recognition process [4]. Developing a stable and reliable AMR system that can operate effectively under such complex conditions is a critical field with significant research value and application prospects.

AMR is essentially a natural text detection and recognition task. A comprehensive understanding of AMR systems requires a systematic analysis of both text-detection and text-recognition components. Text detection, as the initial step in AMR, focuses on localizing and identifying text regions within images. Early research in text detection primarily relied on traditional methods such as Connected Components Analysis (CCA) [5,6,7] and sliding window approaches [8,9] along with Conditional Random Fields (CRFs) [10] and graph-based algorithms [11]. Some of them showed some improvement in challenging scenarios. For instance, Neumann et al. [5] proposed a method based on Maximally Stable Extremal Regions (MSERs), whereas Yin et al. [12] developed a multi-scale feature extraction framework. However, these approaches still faced significant challenges when dealing with extreme lighting conditions, complex backgrounds, and multi-angle text. With the development of deep learning, object-detection-based models have been widely adopted for digital meter region detection. Liu et al. [13] employed Fast R-CNN for meter region detection, whereas Wu et al. [14] achieved high-precision detection through modifications to Mask R-CNN. However, these methods inherited the real-time performance limitations of the R-CNN architecture. To address perspective distortion, Lin et al. [15] combined Polygon-YOLOV5 and YOLOV5s to extract meter regions and reading areas, though this two-step detection approach resulted in suboptimal performance. Peng et al. [16] later proposed the YOLO-CPDM model, an improvement on YOLOV5, achieving a better balance between real-time performance and detection accuracy, yet still showing limitations under extreme lighting variations and image distortion. Some researchers have attempted to enhance model robustness through image preprocessing and data augmentation. For instance, Lin et al. [15] incorporated FFT and DeblurGAN for blur removal during preprocessing, whereas Hou et al. [17] evaluated YOLOX trained with data augmentation across various extreme conditions. However, most of these studies are confined to object detection models or corner detection methods, lacking in-depth research on detecting irregular boundaries. This gap led some researchers to explore scene text detection models, such as Carvalho et al. [18] fine-tuning the EAST model for digital water meter detection and Zheng et al. [19] applying DBNet to X-ray weld image detection, though few studies focused on structural improvements specifically for extreme meter reading scenarios. These studies indicate that relying solely on either object-detection-based algorithms or scene text detection algorithms is insufficient to overcome challenges such as reading perspective distortion and performance degradation in extreme scenarios [20].

Following text detection, the subsequent challenge lies in text recognition, which transforms the detected text regions into characters. Traditional text recognition approaches can be broadly categorized into feature-based methods and sequential decomposition methods. Feature-based approaches utilize direct feature extraction through character segment-based algorithms [21] and label embedding techniques [22], whereas the sequential decomposition paradigm breaks recognition into sub-problems, including text binarization [23], character segmentation [24], and word correction [25]. These approaches shared common limitations in their heavy dependence on handcrafted features, which had shown limited performance on challenge scenarios such as curved text or complex image backgrounds. The introduction of deep learning methods significantly enhanced text recognition performance. Some researchers employed object detection models for digit recognition, such as Liang et al. [26], using YOLOV3-based segmentation and Martinelli et al. [27], improving segmentation recognition performance with YOLOV5s. Peng et al. [16] proposed the YOLO-EERRM model by integrating CBAM attention mechanisms into YOLOV5’s C3 structure, achieving state-of-the-art performance on private datasets. However, object detection methods typically require integration with segmentation algorithms for complete reading recognition, resulting in complex post-processing and potential segmentation errors [20]. In contrast, scene text recognition algorithms are more suitable for automatic meter reading tasks, offering end-to-end recognition of complete text sequences. Some researchers [18,28,29] employed CRNN or its variants (such as LSTM) for digit recognition. Li et al. [29] enhanced recognition performance by embedding CBAM modules in LSTM, whereas Zhang et al. [19] significantly improved performance by incorporating CA and CBAM modules into the MASTER model. These studies demonstrate that scene text recognition methods show greater potential and better text recognition capabilities for meter reading tasks compared to object-detection-based models.

In summary, current automatic meter reading approaches face several critical limitations: (1) Object-detection-based methods typically rely on rectangular or four-points polygon anchor boxes, which are inherently inadequate to capture the irregular and asymmetrical reading regions in AMR tasks [30], leading to significant prediction deviations under perspective distortions. (2) Most robustness improvements focus on extensive data preprocessing such as image enhancement, normalization, and aggressive data augmentation during training, which often compensate for rather than address the intrinsic shortcomings of the model architectures themselves in challenging scenarios. (3) Object-detection-based approaches for digit recognition often require complex post-processing and are prone to segmentation errors. (4) Existing methods typically address either detection or recognition challenges separately, lacking an integrated approach for both tasks. These limitations highlight the need for an end-to-end framework that leverages the strengths of scene text detection and recognition algorithms to handle the unique challenges of meter reading in natural scenes, particularly under extreme conditions [4].

Based on the limitations of existing research, the main contributions of this work are as follows.

(1) A novel and robust AMR framework, EDPNet, is proposed, which integrates scene text detection and recognition algorithms instead of traditional object-detection-based methods. The framework achieves state-of-the-art performance on challenge scenarios compared with methods lacking specific optimization on meter detection and recognition.
(2) EDNet, an excellent detection network replacing backbone with EfficientNetV2-s and incorporating EMA and MSKA attention mechanisms, is proposed. The network achieves significantly enhanced and superior detection results on multiple challenge scenarios, especially irregular reading regions and perspective distortion.
(3) EPNet, an improved recognition network that integrates DropKey attention mechanisms into vision transformer encoders of PARSeq, is proposed. This design enhances recognition performance while effectively mitigating overfitting issues.
(4) Through extensive comparative and ablation experiments, the effectiveness and generalization capabilities of the proposed EDPNet framework across diverse and challenging automatic meter reading scenarios are validated. These results demonstrate that the proposed network achieves an excellent balance between real-time performance and precision while significantly reducing parameters.

The remainder of this paper is organized as follows. Section 2 briefly introduces the principles and implementation details of the proposed method. Section 3 presents the experimental setup in detail. Section 4 provides comprehensive comparative experiments and ablation studies, demonstrating that the proposed method achieves state-of-the-art performance in both conventional and challenging scenarios, thus validating its feasibility and effectiveness. Finally, Section 5 is a summary and outlook.

## 2. Proposed Method

**Figure 1:**
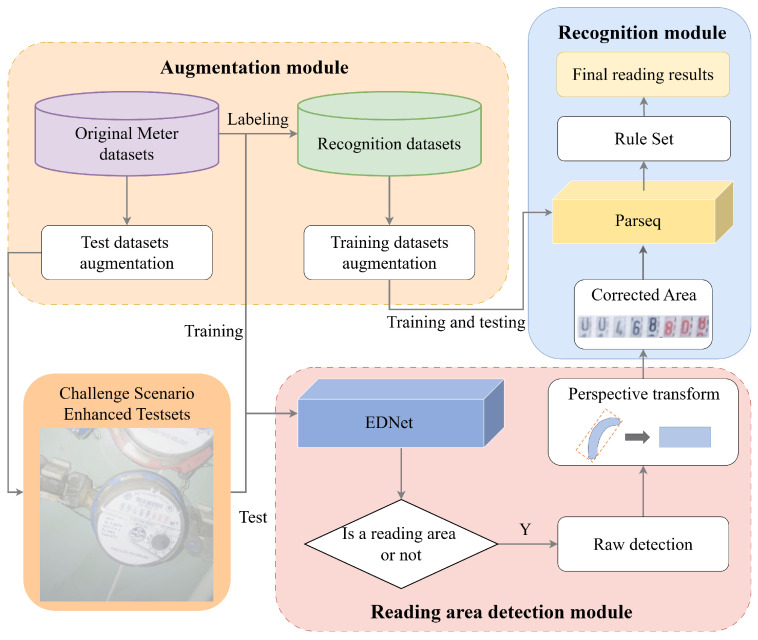
The flowchart for autonomous recognition of digital meter readings.

EDPNet is a real-time digital meter reading system for natural scenes based on deep learning, which integrates EDNet and EPNet. As illustrated in Figure 1, the system’s overall architecture consists of three primary modules: augmentation, reading area detection, and recognition. The augmentation module serves to validate the trained reading area detection module and train the recognition module. By applying data augmentation techniques that simulate challenge scenarios to the original data, the generalization capability and accuracy of the recognition module are effectively enhanced. After the detection module identifies the reading area, perspective-transform algorithms are employed to locate the minimum bounding rectangle of the irregular region and apply perspective correction. The corrected image is then fed into the reading recognition module to produce the final meter reading result. Given the diversity of water meter formats in the dataset, a rule-set mapping is implemented to process decimal points in the recognition results before generating the final output. The subsequent sections provide an in-depth analysis of the architectural design and core methodologies of EDNet and EPNet, highlighting their respective contributions to the overall system.

### 2.1. EDNet

Considering the real-time requirements of water meter reading tasks and the detection characteristics of complex scenarios, this work selects DBNet [31] as the baseline model and makes improvements. DBNet can accurately detect curved text, which is particularly suitable for the common cases of perspective distortion and deformation of reading areas in water meter reading tasks. DBNet mainly consists of three parts: Backbone, FPN neck, and DBHead.

Backbone: The input is a three-channel image. The backbone network extracts features through convolution, generating feature maps $F_{i}\;\left(i=1,2,\cdots ,5\right)$ with resolutions corresponding to 1/2, 1/4, 1/8, 1/16 and 1/32 of original size, respectively, with the number of channels gradually increasing. When selecting different backbone networks, the number of channels in the feature maps may vary; therefore, it is necessary to adjust the configuration accordingly to ensure correct forward propagation.

Neck: Through a top-down process that involves progressive upsampling, residual connections, and convolutional operations, $F_{i}\;\left(i=2,3,4,5\right)$ is reshaped to new feature sets $F_{i}^{\prime }\;\left(i=2,3,4,5\right)\in R^{64\times H/4\times W/4}$, which are subsequently concatenated to form the fused feature map $F_{fpn}\in R^{256\times H/4\times W/4}$

(1) $$F_{fpn}=\mathrm{concat}\left(F_{2}^{\prime },F_{3}^{\prime },F_{4}^{\prime },F_{5}^{\prime }\right).$$

DBHead:

$F_{fpn}$ is processed through a series of convolutions and transposed convolutions to generate the probability map (*P*) and the threshold map (*T*). These maps are then passed through Differentiable Binarization to obtain the final binary map. The operations can be represented as

(2) $$\mathrm{Conv}=\mathrm{ReLU}\left\{\mathrm{BatchNorm}\left[\mathrm{Conv}2d\left(F_{fpn}\right)\right]\right\}.$$

(3) $$P=\sigma \left[\mathrm{ConvTranspose}2d\left(\mathrm{Conv}\right)\right].$$

(4) $$T=\sigma \left[\mathrm{ConvTranspose}2d\left(\mathrm{Conv}\right)\right].$$

(5) $$B=\frac{1}{1+\exp\left[-k\cdot \left(P-T\right)\right]}.$$

where σ represents the Sigmoid function and *k* denotes the amplifying factor for Differentiable Binarization. Though the computation of *P* and *T* are based on $F_{fpn}$, *P* is primarily used to generate the probability distribution of text regions, while *T* is mainly used to generate a threshold map that aligns with *P*, facilitating subsequent binarization. DBNet offers significant advantages over traditional binarization methods. Traditional approaches typically rely on fixed thresholds or global probability distributions, which struggle to handle complex backgrounds, irregularly shaped text regions, and geometric distortions in images. In contrast, DBNet generates a probability map $\left(P\right)$ and a dynamic threshold map $\left(T\right)$, and their combination is used to compute the final binary map $\left(B\right)$. This allows the binarization process to adaptively adjust based on the actual scene. Specifically, the threshold map $\left(T\right)$ can dynamically adjust the binarization standard according to the local information of the input features, improving robustness in complex scenarios.

The adaptive binarization capability of DBNet makes it particularly efficient for region detection in water meter reading tasks. Water meter reading regions in natural scenes often exhibit irregular shapes and are subject to perspective distortion and viewpoint shifts due to varying shooting angles and lighting conditions. The dynamic threshold mechanism of DBNet allows for robust adaptation to varying regional characteristics, enabling the efficient detection of irregular areas. Furthermore, its robust boundary segmentation capability ensures high detection accuracy even in complex scenes, providing reliable technical support for water meter readings.

To address the challenges posed by geometric distortions (including angular distortion and perspective shifts) and adverse imaging conditions (e.g., pixelation, foggy blur, or extreme lighting) frequently encountered in water meter reading tasks, this study proposes EDNet, an efficient detection model built upon the architecture of DBNet.

**Figure 2:**
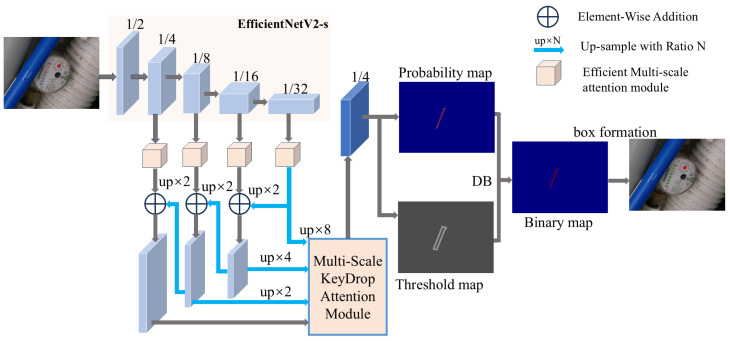
The structure of EDNet.

The overall structure of EDNet is shown in Figure 2. EDNet achieves significantly enhanced detection performance through architectural modifications. Specifically,

(1) The backbone network is replaced with the lightweight yet high-performance EfficientNetV2-s to improve feature extraction in challenge scenarios. This substitution is particularly effective because DBNet’s foundation architecture excels at detecting irregular text boundaries [31], and the proposed EDNet leverages this strength while enhancing it with a superior compound scaling method of EfficientNetV2-s. EfficientNetV2-s systematically balances network depth, width, and resolution, resulting in more robust feature representation and excellent detection performance, particularly for perspective-distorted or irregular meter readings.
(2) The Efficient Multi-scale Attention (EMA) module introduced between the Backbone and Neck addresses the critical issue of feature degradation in foggy and low-contrast environments. By efficiently processing multi-scale feature maps, EMA performs adaptive recalibration of channel-wise features while maintaining computational efficiency. This mechanism specifically enhances the network’s capability to distinguish meter readings from foggy backgrounds by amplifying discriminative feature regions while suppressing irrelevant environmental noise. The multi-scale approach ensures that both fine-grained details (critical for digit recognition) and broader contextual information (essential for boundary detection) are preserved even under challenging visibility conditions.
(3) A Multi-Scale KeyDrop Attention (MSKA) module is proposed. This module integrates channel attention, spatial attention, and enhanced attention mechanisms to hierarchically optimize feature maps, focusing on critical regions. By inserting MSKA between the Neck and Head, EDNet prevents overfitting to specific lightning condition, thus enhancing the capability to tackle extreme illumination challenges in real-world meter reading tasks.

#### 2.1.1. EfficieNetV2

EfficientNetV2, proposed by the Google team at CVPR 2021 [32], is an efficient backbone network that further optimizes training speed and parameter size compared to its predecessor, EfficientNet, achieving significant improvements in multiple performance metrics. EfficientNetV2 adopts a modular stacked design and incorporates dilated convolutions and optimized residual connections, greatly enhancing training efficiency. Experiments demonstrate that EfficientNetV2 achieves an 11-fold increase in training speed and reduces parameters to 1/6.8 of the original size while achieving a higher Top-1 accuracy on the ImageNet dataset compared to traditional backbone networks. Although its inference latency is slightly higher, optimizations for CPU and mobile devices make it superior to traditional backbone networks for mobile deployments [33]. The stacked design of EfficientNetV2 is challenging to visualize directly. Therefore, the detailed structure of EfficientNetV2-s used in this study is presented in Table 1.

In the official release, DBNet++ [34] often employs ResNet or MobileNet variants as backbone networks. However, these networks may struggle to effectively extract features in complex scenarios encountered in meter reading tasks, such as pixelation, foggy blur, and extreme lighting conditions. In contrast, EfficientNetV2 demonstrates outstanding performance in feature extraction and fusion, due to its advanced multi-scale feature pyramid structure and built-in SE attention mechanism. Additionally, its efficient training strategy significantly reduces model training time and resource requirements, making it highly suitable for resource-constrained environments.

**Figure 3:**
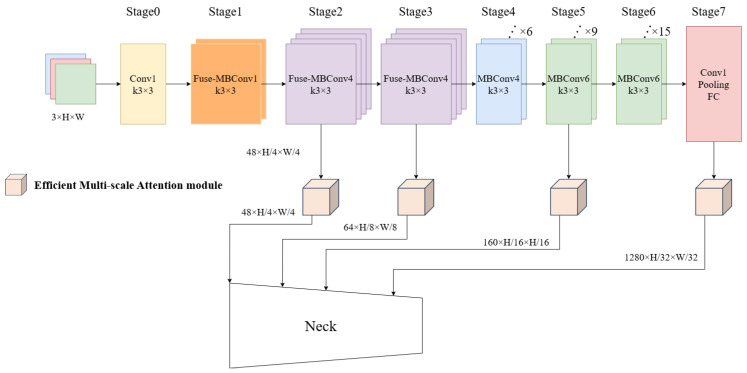
EfficientNetV2-s backbone network.

Based on these advantages, this study incorporates EfficientNetV2-s as the backbone network to further enhance DBNet. As shown in Figure 3, the input image $I\in R^{3\times H\times W}$ is processed through multiple stages in EfficientNetV2-s, where $Stage_{i}$ represents the i-th $Stage\;\left(i=0,1,\cdots ,7\right)$. Specifically, when *i* = 2, 3, 4, 5, the output feature maps are denoted as

$F_{2},F_{3},F_{4},F_{5}$ with channel dimensions of 48, 64, 160, and 1280, respectively. Instead of being directly fed into the Neck, $F_{2},F_{3},F_{4},F_{5}$ are first processed by four separate Efficient Multi-scale Attention (EMA) modules. Then the outputs of these EMA modules serve as inputs to the Neck.

In summary, the challenge scenarios imposes greater demands on the feature-extraction capability of backbone networks. Within both image classification and object detection domains, EfficientNetV2-s outperforms conventional backbone architectures while notably reducing parameters. The modular stacked design and optimized training strategy of EfficientNetV2 enable it to maintain an excellent balance between accuracy and light weight, which serve as two key criteria of meter reading.

#### 2.1.2. Efficient Multi-Scale Attention Module

**Figure 4:**
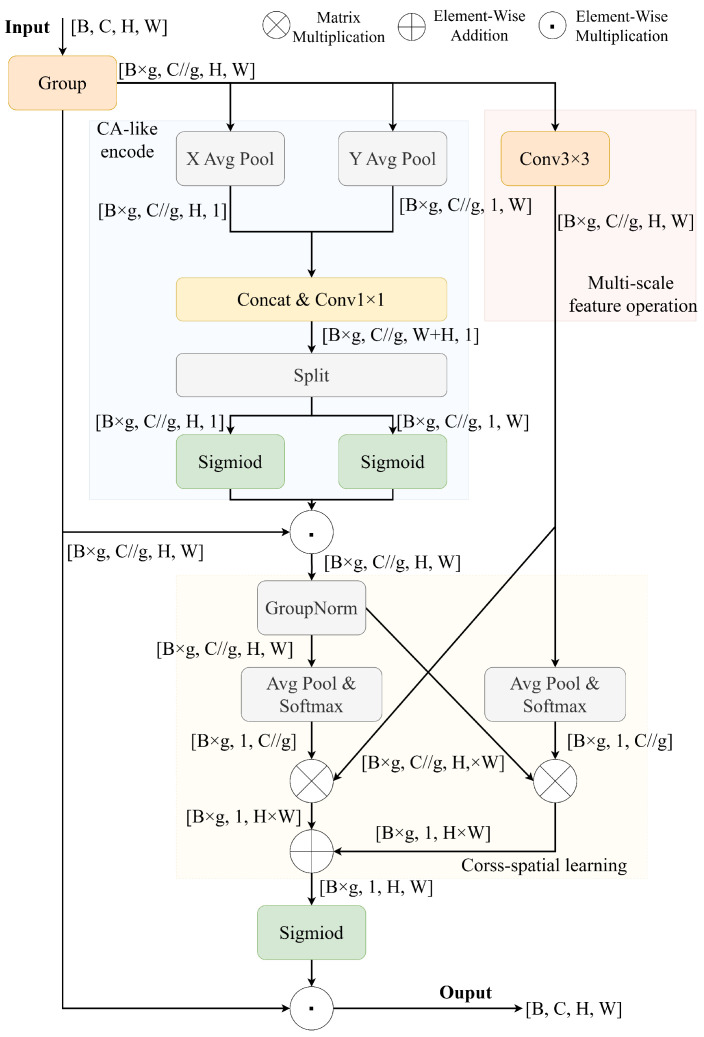
The structure of the EMA module.

To address common challenges in water meter reading tasks, including pixelation and foggy blur of the image, an Efficient Multi-scale Attention (EMA) [35] module is introduced. As shown in Figure 4, the EMA module builds upon Coordinate Attention and enhances its performance. Unlike traditional methods, which use convolution for channel dimension reduction, EMA reconstructs partial channels into batch dimensions and groups channel dimensions into multiple sub-features, thereby avoiding information loss from dimensionality reduction while significantly reducing computational overhead. Specifically, EMA comprises three parallel branches: two branches are computed along the width and height directions of the feature maps, respectively, capturing long-range dependencies; the third branch introduces 3 × 3 convolution to capture local cross-channel interactions, enhancing feature representation capabilities. The outputs from these three branches are fused through a cross-spatial learning strategy to generate the final attention weights.

The EMA module is applied to the four feature maps extracted by EfficientNetV2-s to enhance key feature responses while preserving their original resolution. Unlike conventional attention mechanisms such as CA, SA, CBAM, and ECA, which typically rely on convolution for channel reduction, EMA employs grouped channel reorganization to avoid information loss from dimensionality compression. This design reduces computational overhead and preserves rich channel interactions, making it well-suited for lightweight, real-time models.

Besides the structural design, the choice of EMA is further based on extensive comparisons in its original study [35], where it outperformed the attention mechanisms mentioned above across various tasks. Its ability to model multi-scale dependencies makes it particularly effective in addressing challenges such as pixelation, foggy blur, and geometric distortion, which are common in meter reading scenarios. By improving feature representation with minimal cost, EMA enables a better balance between detection accuracy and inference efficiency. The feature maps processed by EMA are then fed into the Neck for further feature fusion, providing more refined feature input to DBHead, thus improving the model’s performance in automatic meter reading tasks.

#### 2.1.3. Multi-Scale KeyDrop Attention Module

To enhance DBNet’s performance in meter reading detection tasks, particularly in challenging scenarios involving pixelation, foggy blur, and extreme lighting, an MSKA module is proposed. MSKA is designed to enhance the feature processing capabilities of DBHead by integrating channel attention, spatial attention, and DropKey-based enhanced attention mechanisms, hierarchically optimizing feature maps to generate high-quality probability maps.

**Figure 5:**
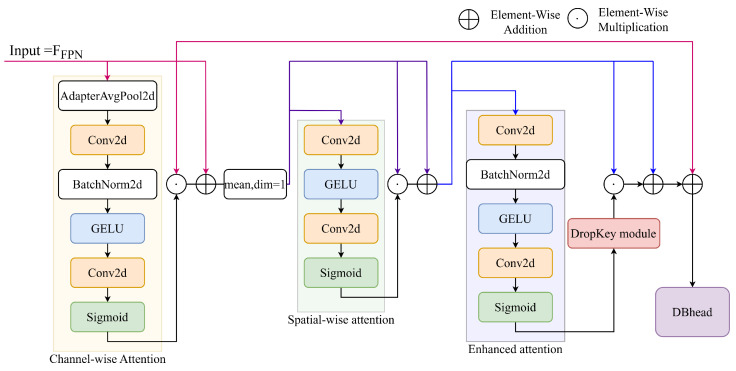
The structure of MSKA.

The structure of MSKA is illustrated in Figure 5,where different colors represent the initial data used for residual addition at different stages. Taking the output from Neck as input $\left(x=F_{fpn}\right)$, MSKA first aggregates global cross-channel information through a channel attention mechanism. It extracts channel features using global average pooling and adjusts channel weights through a lightweight convolutional network while preserving original feature information through residual connections. Subsequently, the spatial attention mechanism focuses on the spatial dimensions of feature maps, generating spatial features through channel averaging and applying convolution operations to highlight key spatial regions, effectively capturing the spatial arrangement information of the meter reading area. These two attention mechanisms work in concert to initially enhance feature representation.

To further improve the performance of the model and reduce overfitting, MSKA incorporates a DropKey-based enhanced attention mechanism. This mechanism draws inspiration from DropKey [36], applying probabilistic masking operations during the training phase to features that have been integrated with channel and spatial attention, simulating a feature regularization process. DropKey encourages the model to attend to different attention regions by randomly dropping certain attention values, reducing dependence on specific dominant features and thereby enhancing model generalization capability. Specifically, given input feature maps x, the DropKey computation is formulated as

(6) $$\text{attn\_map}=\sigma \left(\mathrm{enhance\_attention}\left(x\right)\right).$$

(7) $$mask∼\mathrm{Bernolli}\left(1-\mathrm{dropkey\_ratio}\right).$$

(8) $$mask_{i}=\left\{\begin{array}{cc} 1 & \mathrm{with}\;\mathrm{probability}\;1-\mathrm{dropkey\_ratio}\\ 0 & \mathrm{with}\;\mathrm{probability}\;\mathrm{dropkey\_ratio} \end{array}\right.$$

(9) $$out=F_{fpn}+\text{attn\_map}.$$

where *attn_map* denotes the initial attention map derived from the enhanced attention module, and *mask* denotes the probabilistic masking generated based on Bernoulli distribution and the fixed dropkey ratio. The output attention map serves as input to the DBHead for generating the final probability map.

The core design of the MSKA module aims to enhance feature representation and mitigate overfitting. The former is achieved by integrating both channel-wise and spatial-wise attention mechanisms, while the latter is achieved through the DropKey-based enhanced attention strategy. Although the feature maps produced by the backbone and FPN contain rich information, under challenging scenarios, the model may misinterpret noisy signals as meaningful features. Therefore, MSKA’s joint design of feature reinforcement and feature dropout strategy play a critical role in improving the model’s robustness and generalization in various challenge scenarios.

#### 2.1.4. Loss Function

Since EDNet is based on DBNet, it employs the same loss function as DBNet. The loss function is formulated as

(10) $$L=L_{s}+a\times L_{b}+\beta \times L_{t}.$$

where *a* and β are empirically set to 5.0 and 10, respectively. The total loss function *L* is represented as a weighted sum of the probability map loss $L_{s}$, binary map loss $L_{b}$, and threshold map loss $L_{t}$. Binary Cross-Entropy (BCE) loss is applied to supervise both $L_{s}$ and $L_{b}$, whereas $L_{t}$ is supervised by $L_{1}$ Loss.

### 2.2. Perspective Transformation

To address common challenges of shooting angle deviation and perspective distortion in meter images, OpenCV-based perspective transformation is employed to correct the polygonal regions output by the EDNet model. Specifically, the minimum bounding rectangle for each polygonal region is computed to effectively delineate the target area. The minimum bounding rectangle is chosen because it encompasses the target region with minimal area, thereby reducing interference from background noise. Subsequently, a perspective transformation matrix is calculated using the four vertices of this rectangle to transform the irregular quadrilateral region into a standardized horizontal rectangle. This step effectively corrects geometric distortions caused by shooting angles, presenting the meter region in a standardized form, thus significantly improving the accuracy and robustness of subsequent reading recognition.

Assuming $P_{i}=\left(x_{i},y_{i}\right),i=1,2,3,4$ represent the four vertices of the minimum bounding rectangle, and $Q_{i}=\left(x_{i}^{\prime },y_{i}^{\prime }\right),i=1,2,3,4$ are their corresponding vertices after perspective transformation, the mapping relationship is represented as

(11) $$\left[\begin{array}{c} x_{i}^{\prime }\\ y_{i}^{\prime }\\ 1 \end{array}\right]=M\cdot \left[\begin{array}{c} x_{i}\\ y_{i}\\ 1 \end{array}\right],\;i=1,2,3,4.$$

where *M* denotes the perspective transformation matrix. By applying perspective transformation, distorted regions are corrected into standardized rectangles, effectively improving the accuracy of recognition algorithms in automatic meter reading tasks.

### 2.3. EPNet

PARSeq [37] is an advanced permuted autoregressive sequence model for scene text recognition that achieves efficient processing without relying on external language models. Its robust architecture demonstrates exceptional capability in handling challenging scenarios such as image blur, text curvature, and angular rotation, making it particularly suitable for complex text recognition in meter reading tasks. Unlike traditional approaches that depend on standalone language models for prediction refinement, PARSeq’s efficient design enables superior performance in resource-constrained environments while maintaining high recognition accuracy.

In this study, water meter reading images corrected through perspective transformation are fed into the PARSeq model for reading recognition. To further enhance the model’s performance in automatic meter reading tasks, EPNet is proposed, which improves the PARSeq model by incorporating the DropKey mechanism [36] in the encoder of Vision Transformer. This mechanism randomly drops attention keys with a probability *p* during training to reduce overfitting and improve the model’s generalization ability. Thus, EPNet enhances the focus on key features, making it better suited for handling the diverse text inputs and complex image backgrounds encountered in automatic meter reading tasks. Let the Query, Key, and Value be denoted as $Q\in R^{n_{q}\times d_{k}},\;K\in R^{n_{q}\times d_{k}},\;V\in R^{n_{q}\times d_{k}}$ with a scaling factor of $\sqrt{d_{k}}$. The attention score computation is represented as

(12) $$A=\frac{QK^{T}}{\sqrt{d_{k}}}.$$

where *A* denotes the attention score matrix. *A* random binary matrix *B*, generated according to the Bernoulli distribution and specified dropkey rate, is applied to perturb *A* to obtain the perturbed attention matrix $A^{\prime }$.

(13) $$A^{\prime }=A+B\cdot \left(-1.0e^{12}\right).$$

The normalized attention score matrix *a* is obtained by passing $A^{\prime }$ to the softmax function. During training, Dropout is additionally applied to *a*, further improving the model’s generalization ability. Finally, the output *y* is obtained by performing matrix multiplication with the Value matrix *V*.

(14) $$a=\mathrm{softmax}\left(A^{\prime },\dim=1\right).$$

(15) y=a×V.

EPNet improves recognition accuracy and robustness by incorporating the DropKey mechanism into PARSeq’s Encoder, enabling better adaptation to diverse text inputs and complex image backgrounds encountered in meter reading tasks.

Traditional water meters typically comprise digital readings and pointer readings. Due to various meter designs in the market, the length of numerical digits and the position of decimal points are not standardized. Unlike smart water meters with electronic displays, traditional water meters have their decimal points permanently marked during the molding process. In contrast to YOLO-based recognition methods, EPNet offers the advantage of outputting complete numerical strings. Based on the survey of numerical digits and decimal point configurations in mainstream water meters, mapping is developed between digit count and decimal point positions to automatically correct PARSeq’s output by inserting decimal points at appropriate locations. Specifically, a rule set is designed for the model, which is shown in Table 2. The X and Y represent integers and decimals, respectively.

## 3. Experiment

### 3.1. Experimental Platform and Dataset

**Figure 6:**
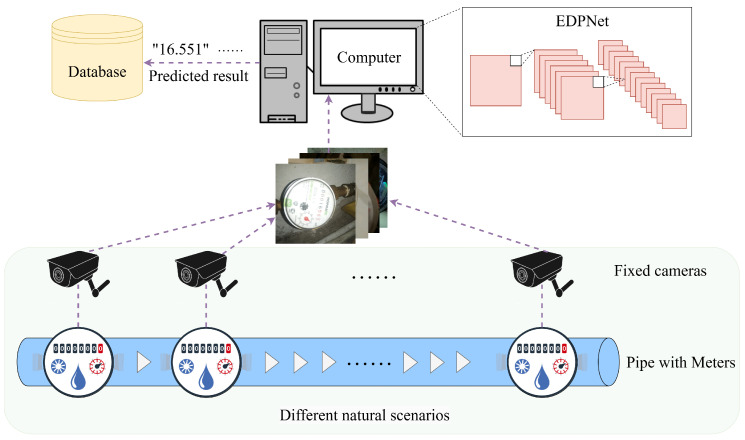
Data flow diagram of the AMR measurement system.

To validate the feasibility of our proposed method, a proof-of-concept experiment aligned with real-world scenarios is conducted using a complete measurement system. Figure 6 shows the data flow diagram of the measurement system, which comprises pipes, meters, fixed cameras, a computer, and a database. Fixed cameras captured the meters under various natural scenarios and transferred the images to the computer. The real-time recognition results predicted by EDPNet based on these images are stored in the database. Following this measurement process, the water meter datasets [38] can be obtained to train and validate the proposed system, which effectively represents the challenges in real-world meter reading scenarios.

**Figure 7:**
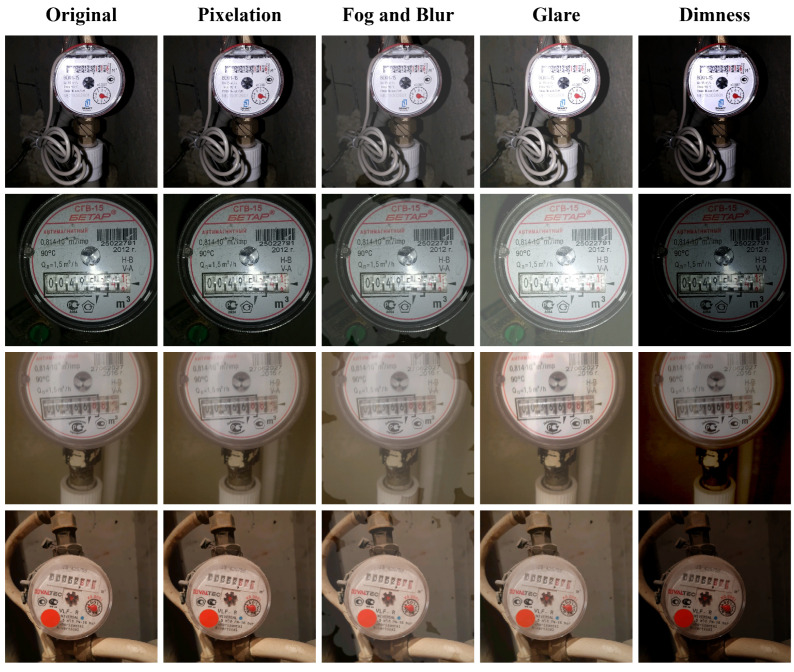
Comparison of the original and augmented test sets.

The detection datasets are generated based on the Water Meters dataset [38]. The original Water Meters dataset contains 1244 images, each featuring a single water meter instance. The dataset is divided into a training set (955 images) and a test set (249 images) at a 4:1 ratio. The training set is augmented using the following strategies: random horizontal flipping, small-angle random rotation (−10° to 10°), and random scaling (0.5× to 3.0×). To evaluate the model’s generalization performance under challenge conditions, several types of offline augmentations are applied to the test set, simulating scenarios such as pixelation, foggy blur, glare, and dimness. The examples of the original test images alongside the augmented test images are presented in Figure 7, demonstrating the four types of data augmentations mentioned above.

**Figure 8:**
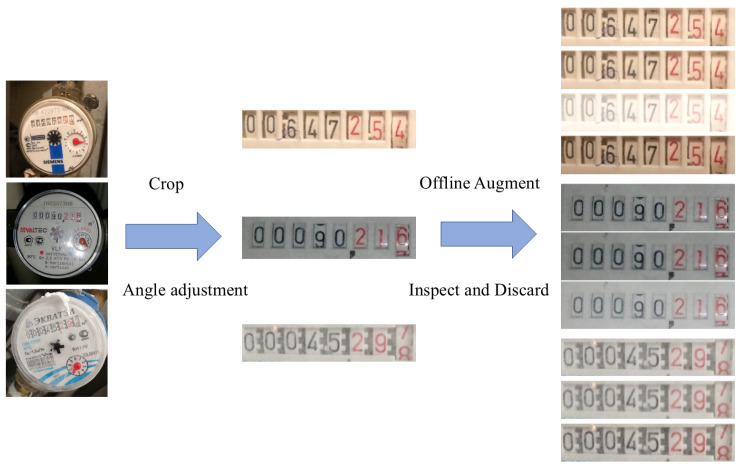
Construction process for the recognition dataset.

To construct the text recognition dataset, the corresponding reading regions are cropped from the Water Meter dataset using the ground-truth bounding box coordinates. These cropped regions are then angle-corrected to ensure the reading areas are horizontally aligned. Next, offline data augmentations are applied to the cropped images using the same strategies as the test set for the detection task above. These include pixelation, foggy blur, glare, and dimness. Each original sample is augmented to generate four enhanced versions, from which unreadable samples are manually removed, resulting in a final dataset of 5228 samples. To prevent data leakage, the dataset is split based on the original image IDs, ensuring that all copies of the same image appear in only one subset. The final dataset is divided into training, validation, and test sets at an 8:1:1 ratio. The construction process of the text recognition dataset is illustrated in Figure 8, including cropping, angle correction, data augmentation, and sample displaying.

### 3.2. Training Details

The experiments are conducted in a Windows 10 environment using an RTX2080Ti GPU, and the project is developed using PyCharm IDE. The detailed experimental environment configuration is presented in Table 3.

The training details for detection: EDNet is proposed for water meter detection. After the model converged on the training sets, it is evaluated on the original test set and four augmented test sets, respectively. The training process employed the stochastic gradient descent (SGD) optimizer with a momentum of 0.9 and a weight decay of 0.001. The learning rate followed a cosine annealing schedule, starting at 0.007 and decaying to 2.6 $\times \;10^{-5}$. The amplifying factor *k* for Differentiable Binarization is set to 50 empirically. Training images are resized to 640 × 640, whereas test images are resized to 800 × 800. The batch size is set to 8, and the number of total epochs is set to 500. The training details for recognition are as follows. Due to the large dataset requirements for training text recognition models from scratch, a transfer learning strategy is adopted and fine-tuned the EPNet on the recognition dataset. Input images are uniformly resized to a fixed size of 32×128. The Adam optimizer is used for training, with $\beta_{1}=0.9$ and $\beta_{2}=0.999$. The initial learning rate is set to $7.7\times 10^{-4}$, with a dropkey ratio of 0.05 and a dropout rate of 0.15. The character set is set to “0123456789”, enabling the recognition of numeric characters only. The maximum label length is set to 25. The batch size is set to 398 and fine-tuned over 20 epochs.

## 4. Results and Discussion

Since the evaluation metrics employed in this experiment are widely known and commonly applied in the field, their definitions and formulas are not elaborated here. Furthermore, the accumulation and prediction of errors due to environmental conditions and inherent instrument limitations fall beyond the scope of this paper, as these aspects have been investigated by other researchers [39,40].

### 4.1. Comparison Result of Detection

**Figure 9:**
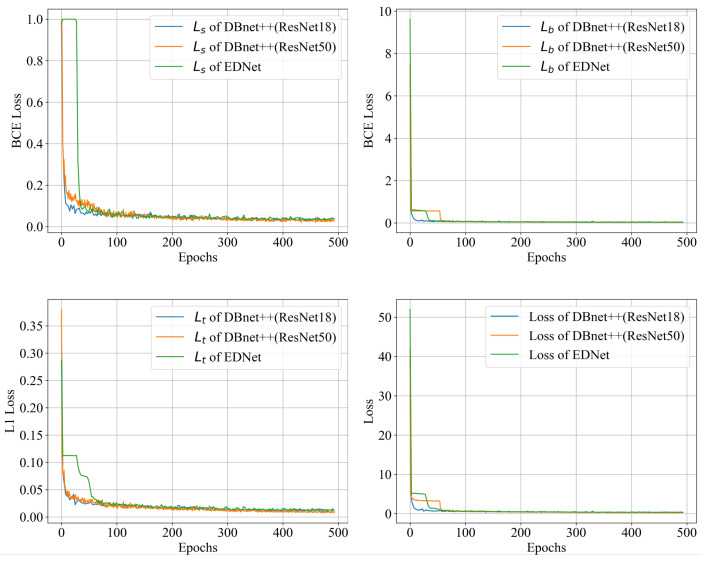
Loss curve of EDNet and DBNet++.

The loss curves during training for EDNet, DBNet++ (ResNet18 or ResNet50) are listed in Figure 9. All three models converge around 100 epochs, with DBNet (ResNet18) achieving the fastest convergence due to its simpler structure and fewer parameters. EDNet demonstrates better convergence speed compared to DBNet++ (ResNet50), indicating more efficient training performance. All models are trained under identical conditions, and their best weights are selected for subsequent performance evaluation.

The detection performances of detection models on the original test set and augmented test sets of four different scenarios are presented in Table 4 and Table 5, respectively. Bold represents the best result, and underline represents the second-best result. This explanation applies to all tables below. Table 4 reports the inference speed of each model in terms of FPSs (Frames Per Second) as well, which serves as an additional metric to evaluate the real-time performance of models. This indicator is solely determined by the model architecture and the number of parameters and is not affected by the datasets.

On the original test set (Table 4), EDNet achieves the best performance. Compared to the SOTA model DBNet++ (ResNet50), EDNet demonstrates improvements of 0.406%, 0.813%, and 0.610% in the precision, recall, and F1-score, respectively, while requiring 20.03% fewer parameters than SOTA. Regarding the real-time inference performance, DBNet++ (ResNet18) achieves the highest speed owing to its simple architecture and fewer parameters. Benefiting from parameter reduction, EDNet achieves the second-best inference performance. These results indicate that EDNet significantly reduces model complexity while keeping an outstanding balance between detection performance and real-time performance.

To evaluate model robustness in challenging scenarios, the performance of different models is further assessed on four augmented test sets (pixelation, foggy blur, glare, and dimness) as shown in Table 5. DBNet++ (ResNet18) and FCENet outperformed DBNet++ (ResNet50) under certain augmented conditions, indicating their generalization capability in specific extreme scenarios. In contrast, EDNet achieved the best F1 scores across all four augmented scenarios, surpassing the second-best models by 1.5%, 0.7%, 1.8%, and 1.9%, respectively. This demonstrates that EDNet exhibits stronger robustness across various challenge scenarios.

**Figure 10:**
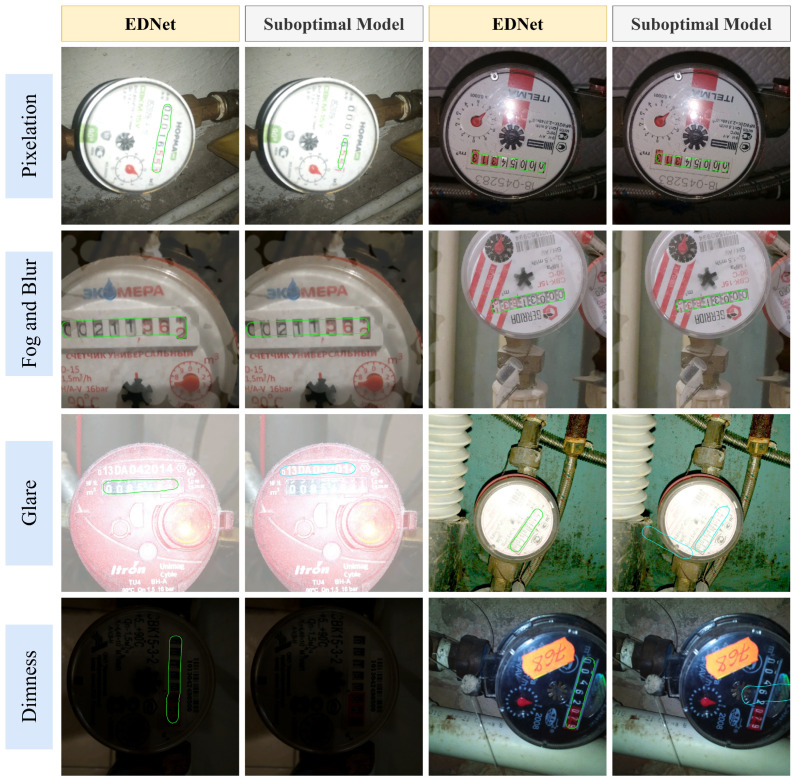
Comparative evaluation of detection models.

The example detection results from different models under four augmented scenarios are illustrated in Figure 10. In relatively simple scenarios such as pixelation and foggy blur, EDNet is able to localize reading regions more accurately, while DBNet++ detected incomplete bounding boxes. EDNet also demonstrated superior precision in boundary handling, capturing more complete display regions, which facilitates subsequent recognition processes. In more challenging scenarios like glare and dimness conditions, FCENet incorrectly identified irrelevant information as reading regions, resulting in erroneous bounding boxes. In contrast, although EDNet was affected by the augmentations, it maintained relatively accurate localization of reading regions.

In conclusion, EDNet not only exhibits excellent performance in standard scenarios but also demonstrates superior generalization capability and robustness across various challenging scenarios. It more effectively generates precise bounding boxes, providing a reliable foundation for subsequent reading area recognition tasks.

### 4.2. Ablation Result of Detection

To thoroughly investigate the interaction between the backbone network, EMA, and MSKA modules and their impact on detection performance, ablation studies are conducted on both the original test set and four augmented test sets (pixelation, foggy blur, glare, and dimness). The ablation results on the original test set are presented in Table 6. The findings indicate the following. Using EfficientNetv2 alone as the backbone network achieves comparable performance to the SOTA model DBNet++ (ResNet50) with an F1-score of 0.991935, demonstrating EfficientNetv2’s strong feature extraction capabilities as a backbone network. The individual implementation of EMA and MSKA modules improves the model’s precision and recall rates (increasing by 0.4% and 0.8%, respectively, compared to the SOTA model), indicating that both EMA and MSKA modules effectively enhance the model’s detection capabilities. When the EMA and MSKA modules are employed simultaneously, the model’s performance improvement is most significant, achieving optimal results in precision, recall, and F1-score (with a 0.61% increase in F1-score compared to the SOTA model). This suggests a synergistic effect between the EMA and MSKA modules in jointly enhancing the model’s detection performance. Furthermore, the EDNet model incorporating both EMA and MSKA maintains high performance while keeping a relatively small parameter count, demonstrating a balance between lightweight design and high performance.

To further validate the effectiveness of the proposed EMA and MSKA modules in challenge scenarios, ablation studies were further conducted on four augmented test sets, with the results shown in Table 7. With the EMA module alone, performance improvements are observed in pixelation and fog scenarios (increases in F1-score of 1.0% and 1.4% compared to the SOTA model). This indicates that the EMA module exhibits a certain robustness to image blur and fog conditions. When using the MSKA module independently, performance improvements were observed across all four scenarios, with the most notable enhancement in glare and dimness conditions (F1-score increase of 1.4% and 1.8% compared to the SOTA model). This suggests that the MSKA module demonstrates strong robustness to illumination variations. When combining both EMA and MSKA modules, optimal performance is achieved in pixel blur, foggy, and high-brightness scenarios (F1-score improvements of 1.2%, 1.6%, and 1.7% compared to the SOTA model), while performance in dimness conditions approached the optimal level (only 0.01% lower than the model using MSKA alone). This further validates the synergistic effect between the EMA and MSKA modules.

These results suggest that the EMA module primarily focuses on image texture and clarity information, hence its significant effectiveness in blur and foggy blur scenarios, while the MSKA module appears to emphasize structural information, resulting in consistent performance across various scenarios, particularly demonstrating strong robustness to illumination variations. The combination of EMA and MSKA effectively utilizes multi-scale and multi-channel information from images, enabling excellent detection performance across various complex scenarios.

### 4.3. Comparison Results of Recognition

As shown in Table 8, the EPNet’s performance is validated on the recognition dataset and compared with other mainstream approaches. In experimental results, EPNet achieved the best accuracy and confidence, with a 0.2% accuracy improvement over the SOTA model to reach 90.0, while the parameter count is maintained at similar levels. These results indicate that our method improves recognition accuracy while preserving model efficiency.

**Figure 11:**
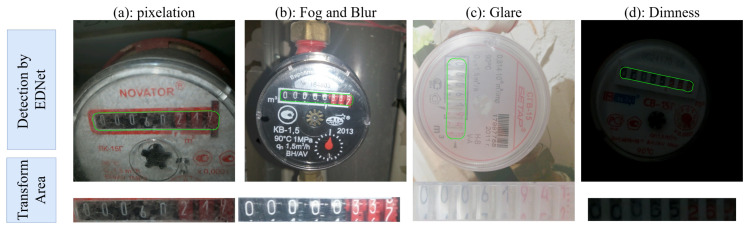
Reading area samples for recognition evaluation.

Four reading region samples detected by EDNet are processed through perspective transformation and shown in Figure 11, corresponding to the four challenging scenarios previously described: pixelation, foggy blur, glare, and dimness. The prediction results from various recognition models are presented in Table 9, where the underline denotes the erroneous recognition result.

The recognition results indicate that CRNN and ABINet exhibited relatively poor recognition accuracy and generalization in these challenging scenarios, frequently producing recognition errors, particularly in pixel blur and fog conditions. VITSTR and TRBA showed better performance but still encountered single-character recognition errors in some samples. PARSeq achieved the second-best performance, with single-character recognition errors occurring only under fog and glare conditions. In contrast, the EPNet model made correct predictions across all four challenging scenarios, demonstrating superior adaptation to extreme conditions and thus better facilitating high-precision meter reading tasks. Combined with the FPS results in Table 8, it can be concluded that EPNet achieved a better balance between recognition accuracy and real-time performance. These results validate the effectiveness of the DropKey attention mechanism in enhancing text recognition model generalization, particularly in challenging scenarios. By incorporating DropKey in the Encoder, the EPNet could learn more precise patterns of challenge scenarios, enabling the better handling of various challenges.

### 4.4. Analysis of Influencing Factors

**Figure 12:**
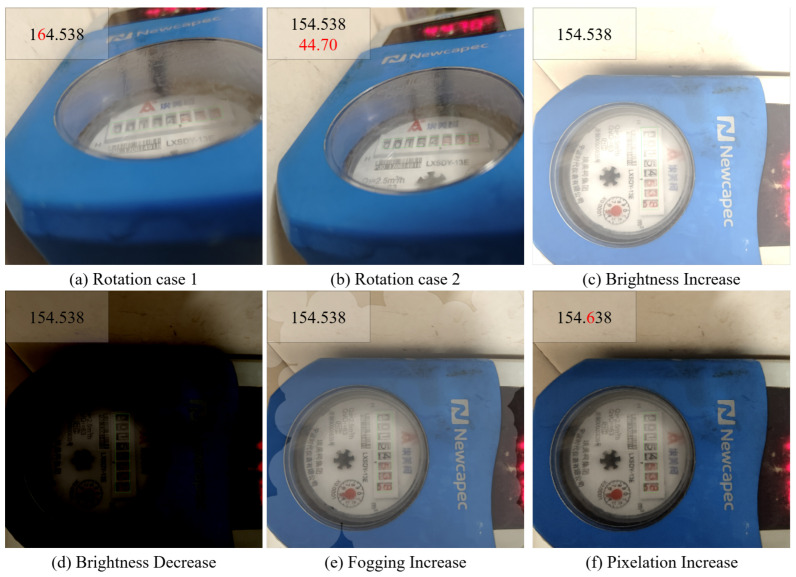
Representative samples obtained from real-world scenarios.

To further validate the generalization capability and practical performance of the proposed framework, multiple water meter photographs using mobile devices as supplementary validation samples are captured according to the process of Figure 6. The acquisition rule involved maintaining parallelism between the camera and the meter reading area while rotating clockwise to collect samples with varying angles. The same data augmentation methods, including glare, dimness, foggy blur, and pixelation, were applied to non-angled samples. Representative samples are illustrated in Figure 12, where green anchor boxes denote detection results of EDNet, top-left annotations indicate recognition results of EPNet, and red markings highlight erroneous predictions. The detection results reveal that EDNet achieves precise localization of the reading area under most challenging scenarios. However, significant angle rotation, like Figure 12b, may occasionally trigger false detections in adjacent regions. Meanwhile, EPNet maintains robust recognition performance even in visually ambiguous scenarios requiring meticulous human inspection, particularly under extreme illumination, foggy, and pixelation conditions.

To systematically investigate the correlation between framework performance and varying interference levels, repeated statistical analyses were conducted using real-world captured samples. Evaluations included different rotation angles, brightness intensities, fogging intensities, and pixelation intensities. Given the limited sample size compared to the original dataset, Max IoU (Intersection over Union) was employed as the evaluation metric for EDNet to quantify detection accuracy of valid regions. For EPNet, error rate calculations followed the methodology explained in Section 4.3, defined as the ratio of misclassified digits to total digits.

**Figure 13:**
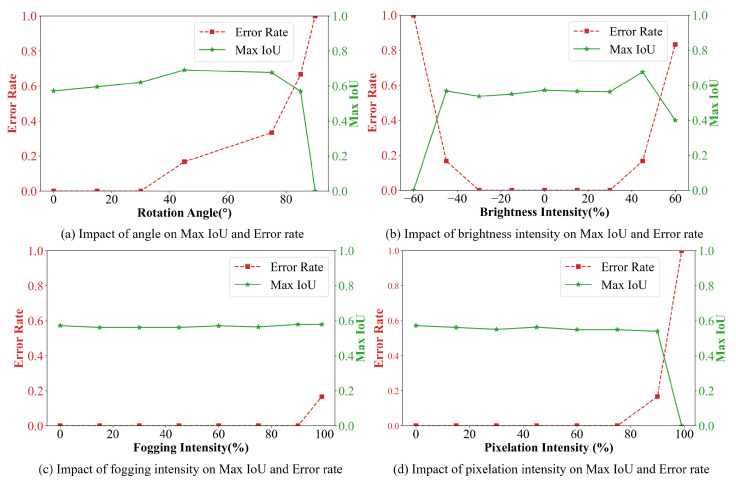
Impact of factors on error rate and Max IoU.

Figure 13 shows the trend curves between the intensities of four influencing factors and the corresponding Max IoU and error rate, which are helpful to evaluate the performance limit and error patterns of the model.

Figure 13a demonstrates the variation curves of Max IoU and error rate across rotation angles. The Max IoU metric exhibits no significant degradation until reaching a critical angle of approximately 75°, beyond which it declines rapidly due to the increasing distance between the camera and meter reading plane, which induces geometric distortions. The error rate shows progressive escalation starting from a critical angle of around 45°, aligning with practical expectations since the input of EPNet depends on EDNet. Based on the curve patterns, an analysis is conducted on Figure 12a,b. In Figure 12b, a smaller rotation angle is observed compared to Figure 12a, which enables more accurate extraction of the reading region. Due to the limited generalization capability of EDNet, regions containing digit features are mistakenly identified as reading regions. However, this misidentification did not interfere with EPNet’s ability to correctly recognize all characters within the actual reading area. In contrast, Figure 12a exhibits a larger rotation angle, which leads to an increased recognition error. This finding aligns with the statistical trends presented in Figure 13. Experimental results confirm that the EDPNet framework maintains superior recognition accuracy (<45° rotation) in real-world scenarios, demonstrating effective angle distortion robustness.

Figure 13b presents the variation curves under varying brightness conditions, where negative/positive values indicate reduced/enhanced brightness ratios. Both dimness and glare scenarios exhibit critical thresholds at around 45% intensity. While Max IoU remains stable and error rates stay low within this range, exceeding these thresholds triggers significant performance deterioration. This aligns with practical limitations where human operators cannot reliably discriminate meter readings beyond ±60% illumination as well. Figure 12c,d present examples of increased and decreased brightness, respectively. It can be observed that under relatively extreme brightness variations, EDPNet is still capable of accurately detecting the boundaries and recognizing the correct digit readings. Such variations in brightness are estimated to remain within the threshold range of ±45%. The results confirm EDPNet’s robustness against extreme lighting conditions in realistic application scenarios.

Figure 13c analyzes fogging simulation effects, revealing distinct behavior compared to angle and illumination challenges. Gradual fogging intensification produces negligible impacts on both metrics, attributable to the simulation’s preservation of semi-transparent features. Figure 12e illustrates a sample under fogging conditions. According to the statistical trends, the fogging intensity in this case is estimated to be no greater than the threshold of 90%. Even under high intensity of fogging, EDPNet sustains stable detection accuracy and recognition reliability.

Figure 13d illustrates pixelation effects, with a critical degradation threshold emerging at around 90% resolution reduction. Beyond this point, error rates escalate rapidly due to the progressive loss of discriminative features in reading regions. Figure 12f shows an example under pixelation. Based on the observed patterns, the degree of pixelation in this instance is estimated to be below the 90% threshold. The framework nevertheless demonstrates remarkable generalization capability, maintaining practical usability across most pixelation levels encountered in real-world deployments.

The experimental findings demonstrate that EDPNet exhibits several representative error patterns across both benchmark datasets and real-world sample evaluations. The summary and analysis are concluded as follows: each error type, separated by semicolons, is correspondingly matched with its cause analysis and a possible solution.

(1) EDNet Errors
  Error Types: Incomplete segmentation boundaries; misidentification of incorrect areas as reading areas; detection failure, unable to obtain reading frames (such as exceeding the thresholds referring to the trend curves mentioned above).
  Cause Analysis: Insufficient detection precision of EDNet; model overfitting, detecting some areas with digits as reading areas; poor input image quality, possibly indiscernible even to human eyes.
  Possible Solutions: Consider using mixed digit character datasets, not just water meter datasets, to help EDNet learn various digit feature patterns; consider filtering out non-reading area anchor boxes based on aspect ratio and other geometric information; confirm and eliminate data that cannot be recognized.
(2) EPNet Errors
  Error Types: Incorrect recognition of one or several digits; missing digits or multiple incorrect outputs.
  Cause Analysis: Insufficient generalization ability and recognition accuracy of EPNet; incomplete segmentation boundaries or multiple incorrect boundaries from EDNet.
  Possible Solutions: Use larger datasets to fine-tune EPNet and enhance recognition performance; use the same improvement strategies as for EDNet.
(3) Rule Set Errors
  Error Types: Incorrect decimal point position.
  Cause Analysis: Error type 2 of EPNet (missing digits or multiple incorrect outputs).
  Possible Solutions: Same solutions as for EPNet.

## 5. Conclusions

In the detection task, EDNet employs EfficientNetV2-s as its backbone network with the proposed Multi-scale KeyDrop Attention (MSKA) module and introduced Efficient Multi-scale Attention (EMA) module, thereby enhancing boundary detection precision and recall, particularly in challenging scenarios such as pixelation, blur, glare, and dimness conditions. The model’s generalization capability and robustness are effectively enhanced through perspective transformation of the detection results. In the recognition task, EPNet, based on the PARSeq framework, incorporates a DropKey Attention module to mitigate overfitting and improve recognition accuracy. Experimental results demonstrate that EPNet achieves a recognition accuracy of 90.0%, surpassing the current state-of-the-art methods by 0.2%. The following conclusions can be drawn:

(1) Superior Detection Performance: EDNet achieves an F1 score of 0.997988 on the original test set, representing a 0.61% improvement over DBNet++. On the four augmented test sets, it demonstrates improvements in accuracy of 0.7~1.9% compared to the existing best methods. Additionally, EDNet reduces parameter count by 20.03% compared to DBNet++ (ResNet50), achieving a superior balance between high precision and lightweight real-time performance.
(2) Enhanced Robustness: EDNet exhibits strong adaptability to complex scenarios involving perspective distortion and rotation. It demonstrates exceptional detection performance in challenging scenarios such as pixelation, foggy blur, glare, and dimness, validating its robustness.
(3) Enhanced Recognition Performance: With the integration of the DropKey Attention module, EPNet significantly improves recognition accuracy to 90.0% without substantially increasing parameters, demonstrating excellent practicality and reliability.
(4) Through statistical analysis with real-world detection data, critical performance thresholds of EDPNet under extreme conditions are found. Typical errors include incomplete segmentation boundaries and digit misrecognition. Performance deteriorates when rotation angles exceed 75° due to perspective distortions causing a rapid IoU decline; brightness variations beyond ±45% trigger significant error rate increases; fogging shows minimal impact even at high intensities owing to preserved semi-transparent features; and pixelation increases over 90% lead to rapid performance degradation from the loss of discriminative features.

In conclusion, the proposed EDPNet demonstrates considerable potential for text extraction and recognition tasks in diverse challenge scenarios, extending beyond digital meter reading to applications such as expiration date detection on food packaging and the automatic parsing of industrial equipment labels. Based on the identified error patterns, future research will focus on two critical directions: (1) Enhancing model robustness at critical thresholds by developing a comprehensive solution that integrates higher-quality composite training datasets with advanced geometric filtering algorithms, addressing both detection limitations under extreme rotational angles (>75°) and illumination variations (±45%), while preserving valid anchor boxes in challenging recognition scenarios. (2) Explore the deployment of the model entirely on mobile devices for real-time automatic meter-reading breakthroughs, which is more emphasized on deployment than the work in this paper.

## Figures and Tables

**Table 1:** The structure of EfficientNetV2-s.

Stage	Operator	Channels	Layers	Stride
0	Conv3 × 3	24	1	2
1	Fused-MBConv1, k3 × 3	24	2	1
2	Fused-MBConv4, k3 × 3	48	4	2
3	Fused-MBConv4, k3 × 3	64	4	2
4	MBConv4, k3 × 3, SE0.25	128	6	2
5	MBConv6, k3 × 3, SE0.25	160	9	1
6	MBConv6, k3 × 3, SE0.25	256	15	2
7	Conv1 × 1 & Pool & FC	1280	1	1

**Table 2:** Rule set.

Length of Digits	Decimal Point Position
8	XXXXX.YYY
7	XXXXX.YY
5	XXXXX.

**Table 3:** Operating environment.

Hardware Configurations	Software Configurations
Operating System: Windows 10	Pycharm Version: 2024.3
GPU: NVIDIA GeForce RTX 2080Ti	Python Version: 3.8, 3.10
CPU: Inter® CoreTM i9-9900K	Python Version: 3.8, 3.10
RAM: 64GB DDR4 2133MHz	cuDNN Version: 8.9.5, 8.9.7

Manufacturer for GPU: Santa Clara, CA, USA; CPU: Santa Clara, CA, USA.

**Table 4:** Comparison results on the original test set.

Model	Params (M)	Precision	Recall	F1-Score	FPS
DBNet++ (resnet18) [34]	**13.96**	0.987952	0.987952	0.983871	**70.7**
DBNet++ (resnet50) [34]	29.00	0.995951	0.987952	0.991935	30.4
FCENet [41]	28.07	0.953120	0.737900	0.831818	17.9
PSENet [42]	28.70	0.979919	0.983870	0.981891	13.0
EDNet (Ours)	23.19	**1.000000**	**0.995984**	**0.997988**	39.8

Bold represents the best result, and underline represents the second-best result. The same applies below.

**Table 5:** Comparison result on the augmented test set.

Model	Pixelation	Fog and Blur	Glare	Dimness
DBNet++ (resnet18)	0.978526	0.974937	0.929745	0.929874
DBNet++ (resnet50)	0.973154	0.988975	0.933962	0.942138
FCENet	0.967239	0.969743	0.941923	0.944257
PSENet	0.965795	0.964000	0.899383	0.909853
EDNet (Ours)	**0.993988**	**0.995984**	**0.959514**	**0.963265**

Evaluation Matrix: F1-score.

**Table 6:** Ablation study on the original test set.

EMA	MSKA	Params. (M)	Precision	Recall	F1-Score
		23.19	0.995951	0.987952	0.991935
✓		23.19	**1.000000**	0.987952	0.993939
	✓	23.19	0.992000	**0.995984**	0.993988
✓	✓	23.19	**1.000000**	**0.995984**	**0.997988**

Evaluation Matrix: F1-score; The ✓ denotes that the corresponding module is incorporated into the model.

**Table 7:** Ablation study on the augmented test set.

EMA	MSKA	Pixelation	Fog and Blur	Glare	Dimness
		0.981325	0.979834	0.943218	0.945327
✓		0.992000	0.994012	0.944020	0.946701
	✓	0.988000	0.983988	0.957404	**0.963415**
✓	✓	**0.993988**	**0.995984**	**0.959514**	0.963265

Evaluation Matrix: F1-score; The ✓ denotes that the corresponding module is incorporated into the model.

**Table 8:** Comparison results of recognition on the test set.

Model	Params. (M)	Accuracy	Confidence	FPS
CRNN [43]	**8.361**	0.6481	0.6423	**245.1**
ABINet [44]	36.86	0.8385	0.8500	41.9
TRBA-Net [45]	49.82	0.8712	0.9020	50.2
ViTSTR [46]	21.70	0.8115	0.8958	131.8
PARSeq [37]	23.83	0.8981	0.9184	89.2
EPNet (Ours)	23.83	**0.9000**	**0.9318**	90.2

**Table 9:** Comparative recognition samples of models.

Sample	Ground Truth	CRNN	ABINet	TRBA	VITSTR	Parseq	Ours
(a)	60.219	80.219	60.213	60.218	40.219	60.219	60.219
(b)	0.337	0.192	69.337	0.357	0.387	0.357	0.337
(c)	64.941	64.947	64.947	64.948	64.949	64.947	64.941
(d)	65.26	65.267	65.264	65.263	65.263	65.26	65.26

The underline represents the erroneous recognition result.

## Data Availability

The original Water Meter dataset used in this study is publicly available at Water Meters Dataset (https://www.kaggle.com/datasets/tapakah68/yandextoloka-water-meters-dataset, (accessed on 28 June 2024)). The derived detection and recognition datasets will be provided upon request to the authors.

## Abbreviations

The following abbreviations are used in this manuscript:

EDPNet	Efficient DB and PARSeq Network
EDNet	Efficient DBNet
EPNet	Efficient Parseq Network
FPN	Feature Pyramid Networks
MSKA	Multi-Scale KeyDrop Attention
EMA	Efficient Multi-scale Attention
AMR	Automatic Meter Reading
YOLO	You Only Look Once
PARSeq	Permuted Autoregressive Sequence
SOTA	State-Of-The-Art
